# Survived but feeling vulnerable and insecure: a qualitative study of the mental preparation for RTW after breast cancer treatment

**DOI:** 10.1186/1471-2458-12-538

**Published:** 2012-07-23

**Authors:** Corine Tiedtke, Angelique de Rijk, Peter Donceel, Marie-Rose Christiaens, Bernadette Dierckx de Casterlé

**Affiliations:** 1 Department of Occupational, Environmental and Insurance Medicine, Katholieke Universiteit Leuven, Kapucijnenvoer 35/5, B-3000, Leuven, Belgium; 2 Faculty of Health, Medicine and Life Sciences, Department of Social Medicine, Maastricht University, P.O. Box 616, 6200 MD, Maastricht, the Netherlands; 3 Department of Occupational, Environmental and Insurance Medicine, Katholieke Universiteit Leuven, Kapucijnenvoer 35/5, B-3000, Leuven, Belgium; 4 Multidisciplinary Breast Centre, Katholieke Universiteit Leuven and University Hospital Leuven, Herestraat 49, B-3000, Leuven, Belgium; 5 Centre for Health Services and Nursing Research, Katholieke Universiteit Leuven, Kapucijnenvoer 35/4, B-3000, Leuven, Belgium

**Keywords:** Qualitative, Breast Cancer, Work incapacity, Mental preparation, Interaction, Social environment, Vulnerability, Return to Work (RTW)

## Abstract

**Background:**

Improvements in treatment have resulted in an increasing number of cancer survivors potentially being able to return to work after medical treatment. In this paper we focus on the considerations regarding return to work (RTW) of breast cancer absentees in the Belgian context and how these considerations are related to reactions from their social environment.

**Methods:**

A qualitative study was performed to understand the RTW considerations of Belgian breast cancer absentees who had undergone breast cancer surgery in 2006. Twenty-two participants (mean age 46) were included and interviewed between May 2008 and August 2009 in their personal environment. An in-depth analysis (Grounded Theory) took place using the Qualitative Analysis Guide of Leuven (Quagol).

**Results:**

Before the actual RTW, breast cancer employees try to build an image of the future resumption of work based on medical grounds and their knowledge of the workplace. Four matters are considered prior to RTW: (i) women want to leave the sick role and wish to keep their job; (ii) they consider whether working is worth the effort; (iii) they reflect on their capability; and (iv) they have doubts about being accepted in the workplace after returning. These inner thoughts are both product and input for the interaction with the social environment. The whole process is coloured by uncertainty and vulnerability.

**Conclusion:**

Our study demonstrated that mental preparation for RTW is not a linear process of improvement. It shows a detailed picture of four types of considerations made by breast cancer survivors before they actually resume work. Vulnerability appears to be an overarching theme during mental preparation. As the social environment plays an important role, people from that environment must become more aware of their influence on decreasing or increasing a woman’s vulnerability while preparing for RTW.

## Background

Early detection and improvements in treatment in the developed countries have resulted in an increasing number of cancer survivors [1], which means that more survivors might be capable of returning to work (RTW). The current study focuses on breast cancer. The burden of breast cancer in Belgium is very high. In 2006 breast cancer incidence among women in the Flemish region of Belgium (Dutch-speaking Belgium) was 5,511 for all ages; 1,158 for the 35–49 age group and 2,570 for the 50–69 age group [2]. Johnsson et al [3] found that about 60 % of the women with breast cancer returned to work 10 months after surgery. In a Dutch sample of breast cancer survivors, Roelen et al [4] found a mean duration of sickness absence of nearly a year (12 % lasted longer than 2 years), but in the end most women returned to work. Other studies for this group pointed at lower RTW rates and reported that they depend on type of treatment [5,6].

This paper focuses on the mental preparation for RTW by breast cancer patients. What do they consider and why? Several theoretical models for return-to-work (RTW), for example the Phase model of Occupational Disability [7], the Readiness for Return-to-Work model [8] or the Attitude, Social norm and self-Efficacy (ASE) model that has been applied to return to work [9], assume that patients differ in the degree to which they are mentally prepared for RTW. Franche and Krause [8] incorporated the original Prochaska et al [10] motivational stages of change (precontemplation, contemplation, preparation, action and maintenance) in their model. During the ‘contemplation’ phase a disabled person is beginning to consider RTW and in the ‘preparation for action’ phase he/she is making concrete plans to return. They defined three dimensions of change in making progress during the several phases: decisional balance (reflects the cognitive process of weighing pros and cons), self-efficacy (refers to one’s confidence in engaging in RTW) and change processes (concerns the perceived need to change and the actual behavioural change).

Earlier studies have demonstrated how breast cancer patients experience the early phases of work disability. Receiving a breast cancer diagnosis causes emotional trauma due to the fear of dying or extremely negative thoughts in preparing for the worst. Women have to employ several strategies to cope with the illness, and might become uncertain about their future quality of life, including work [11-14]. Recently, Tiedtke et al [15] found that patient experiences of being work disabled due to breast cancer can vary greatly. Three groups of experiences were identified in a Flemish (Belgian) sample: (1) a disruption, with feelings of loss and despair; (2) an unpleasant but temporary episode; and (3) a meaningful period after which new life priorities are set. Salander et al [16] found comparable groups in a sample of Swedish women who survived breast cancer.

However, we do not know how the patients themselves experience their preparation for RTW or how they make decisions about RTW after (longstanding) illness. The combined Readiness for Return-to-Work model also elucidates the interpersonal context (workplace, healthcare, and insurance system) of the work-disabled person at the different decisional stages [8]. There is evidence that support in the social context is of significant importance for initiating RTW [11,17,18]. The aim of this paper is therefore to elucidate the experiences of breast cancer patients who consider returning to work after medical treatment and improve our understanding of how these are related to their social environment. The study took place in the specific context of Flanders (Belgium), which lays the emphasis on a compensation policy approach as opposed to a reintegration policy approach [19]. Employers only pay for the first 2–4 weeks of sick leave; after that wages and check-ups are covered by national health care and benefit insurance. The aim of our research is to understand how Flemish (Belgian) breast cancer absentees prepare for their RTW.

## Methods

### Design

We used a qualitative design, based on a Grounded Theory approach [20] to understand the RTW considerations of Belgian breast cancer absentees.

### Data collection

All employees (up to 55 years) who had undergone breast cancer surgery in 2006 (n = 65) were invited to take part in our study by a Belgian health insurance service, from which they received sick leave wages, and twenty-four women responded with informed consent. After contacting them to collect demographical, medical and work details, twenty-two agreed to continue cooperation. Between May 2008 and August 2009 in-depth interviews (n = 22) were performed in the women’s personal environment, mostly at their homes. They lasted for an average of 70 minutes. Open-ended questions were used and we specifically asked for their experiences in preparing for RTW. The main interview questions addressed the employee’s experiences of being at home during breast cancer treatment and initiating or preparing for RTW after recovery as well as the environmental social support (work, medical, insurance, and private environment) experienced when deciding whether or not to return to work. For instance, when you decided that you wanted to return to work after treatment, what did you do; who advised you regarding RTW and what sort of advice did you receive; how and when did you decide whether to return to work or not, what help did you get and from whom; what problems did you encounter and why; how did you feel; what kind of reactions did you get? To ensure the quality of the questions, regular meetings with the research team were held. Comments were integrated in the interview guide, which evolved over time. Interviews were all conducted and transcribed verbatim by the same researcher (CT).

### Participants

We included twenty-two Flemish (Dutch-speaking Belgium) employees (Province of Limburg) who had undergone breast cancer surgery in 2006 and excluded women working for the government and self-employed women (because of different legal arrangements). The mean age at time of surgery was 46 (41–55 years). All women underwent chemotherapy and/or irradiation therapy after mastectomy or breast conservative surgery (almost equally divided) and three women suffered a recurrence in 2006. The participants were high-school graduates and the broad professional categories were service and administration professions: office worker (n = 6), caregiver (n = 10), shop-assistant (n = 6). Half of the women (n = 11) had returned to (former or new) work at the time of the interview, which was after a mean time of 30 months (2.5 years) after surgery. Table 1

**Table 1:** Participant characteristics

	**N = 22**		**N = 22**
*Age*		*Education*	
40-45	6	Low	3
45-50	6	Medium	13
50-55	10	High	6
*Surgery*			
Breast conservative surgery		Adjuvant therapy	
+ axillary dissection	4	Irradiation	4
+ sentinel node procedure	7	Chemo therapy	5
Mastectomy		Irradiation and Chemo	13
+ axillary dissection	10		
+ sentinel node procedure	1		
(+ reconstruction)	(4)		
*Job type*		*Returned to work*	
Office worker	6	Yes	11
Care giver	10	No	11
Shop assistant	6		

### Analysis

After transcribing the interviews, a profound analysis based on the Qualitative Analysis Guide of Leuven (QUAGOL) [21] took place, with constant data comparison and interactive team dialogue about reflections and concepts. A Grounded Theory approach is 1) used to answer the research question, which is about views, meanings and concrete experiences of women with breast cancer, and 2) used to achieve the aim of elucidating the experiences of breast cancer patients who consider returning to work after medical treatment and improve our understanding of how these are related to their social environment.

The QUAGOL is a theory- and practice-based guide that helps researchers to analyse qualitative data using a Grounded Theory approach in a structured, although not rigid way. It was developed to facilitate capturing the rich insights from qualitative data. The strengths of the guide lie mainly in the case-oriented approach characterized by a continual balancing between within-case and cross-case analysis, a forward–backward dynamic using the constant comparative and the interdisciplinary team approach [21]. The process of analysis consists of two parts: (1) a thorough preparation of the coding process, implying only paper and pencil work, and (2) the actual coding process using a qualitative software program.

Preparation of the coding process was made by a thorough (re)reading of the interviews and phrasing the understanding of the interviewee’s story in answer to the research question in a narrative report and/or a conceptual scheme per interview. After that the authors, who all had different expertise (breast cancer surgeon, social scientists, insurance physician) in addition to a (qualitative) research background, verified the schemes regarding content and concepts. If needed, schemes were adapted or refined. The concepts of the interview schemes were tested and developed by means of comparison with schemes and data from other interviews. After refining and comparing, the actual coding process took place by drawing up a list of contextually and analytically meaningful concepts, which were tested and refined again by re-reading all interviews as many times as necessary. Significant passages of the interviews were linked to one of the concepts, using the QSR NVivo 8 program, which is the second step: the actual coding process. Then we identified common messages describing the essence of the concepts and split the concepts if required into several sub-concepts. Finally, we described the results on a conceptual level, grounded in the interview data. All team reflections and discussions (about schemes, concepts, and lists of quotes), were transcribed and used in the analysis process and description of the findings (by the first author). Before summarising the results, they were presented and discussed with a panel of experts, including three members of the team (breast cancer surgeon, social security physician, social scientist), thus enhancing the trustworthiness of the findings. The panel of experts (n = 10) was recruited by the team (convenience sampling) and consisted of five social scientists (experience with qualitative research) from the universities of Leuven (Belgium), Maastricht and Amsterdam (the Netherlands), a nurse practitioner (experienced counsellor of women with breast cancer), a breast cancer surgeon, two social security physicians, and a psychologist (communication expert).

### Ethical considerations

We requested and received ethical committee approval (Ethical Committee of the Faculty of Medicine, Catholic University of Leuven) and the data collection was carried out with care and concern. The privacy and confidentiality of the participants was maintained and therapeutic support was available in case of emotional problems.

## Results

### Inner considerations concerning RTW

Our data showed that, before the women who were treated for breast cancer actually returned to work, they very carefully considered how to make the transition from ‘being ill’ to ‘returning to work’. During the period of work disability they tried to build an image of the future resumption based on medical grounds and their knowledge of the workplace. A process of mental preparation took place, characterised by uncertainty and vulnerability as universal themes Table 2.

**Table 2:** Themes and subthemes regarding inner considerations

**Themes**		**Subthemes**
Away from the sick role and wanting to keep the job	·	Being ill and feeling recovered
	·	Not wanting to be stigmatised as a disabled person
	·	Return to a normal life
	·	Contributing to society
	·	Not wanting to give up their work
Is it worth making the effort to return to the job?	·	Efforts compared to the consequences (financial, medical, and personal)
	·	Uncertain health / fear of recurrence
	·	Individual need to return?
Concerned about recovery	·	Performing as an employee again?
	·	Desire to return to work
	·	Defining capability level
	·	Fearing recurrence
Doubts about acceptance in the workplace	·	Workplace understanding of the employee’s situation?
	·	100 % performance might not be possible
	·	Weighing up capacity and motivation level knowing the workplace
	·	Have to feel strong and in good health to meet the employer

After a short- or long-term recovery at home, the women tried to create a certain image of being at work again. They reflected on various unanswered questions they had. Many of the women wanted to leave the sick role and secure their work role, but wondered whether they had recovered enough to return to their former job, or a new and adapted one. Returning to work might be too difficult and therefore make demands on their (vulnerable) health. How productive and functional would they be? Would they meet the expectations of their employers? The women had inner considerations about different domains related to recovery and capability associated with more or less strong emotions. Their reactions varied as regards content and intensity, i.e. not all women per se experienced all considerations with the same intensity of emotions.

#### Away from the sick role and wanting to keep the job

Being at home felt like being ill, whilst being at work felt like having recovered, according to some interviewees. Although these women mentioned some advantages of being disabled at home, their preference was to work. One woman said she would prefer to have worked for two whole years than to have suffered breast cancer. These women indicated their impatience regarding work resumption, because this would offer an opportunity to leave behind their illness.

*"“… and by the way yes, I had been ill, it was over, I wanted to be my old self again, I didn’t want to be ill anymore, I wanted to participate normally again…”"*[1]

Being at home felt like being in a sort of limbo, not knowing how to proceed with their life, as some women mentioned. They wanted to be their old self again and return to a normal life, which included work, although this would require a lot of them and would take some getting used to again.

*"“… I didn’t want to be sitting at home all the time and I really wished that I could have started that job that I’d got at the time, yes, because I liked doing that. I wanted to try to do it part-time, see if I could handle that (…) I believe I wasn’t the person that I could be, that I had to stay home for whole days because I was ill, I think that was the problem. I didn’t want to be ill and I think that I wanted to return to work so fast (…) that everything would be fine. When I get outside, then my disease is gone, then I will be cured…”"*[15]

The interviewees emphasised that they did not want to give up their work. Some certainly could not do without it and they also felt the need to contribute to society, if necessary part-time or as a volunteer. Furthermore, they said that they did not want to be stigmatised as a disabled person.

*"“… I don’t want to stay at home, I want to be among people, I want to return to work, I want to be busy, I actually still have a future ahead of me, I really am too young: I am disabled, I’m going to be 44 years old, I’m having a hard time with that. I always used to think, I will go to work and everything will be fine, I was never really ill, never at work …”"*[2]

#### Is it a worth making the effort to return to their job?

Secondly, the women considered the efforts compared to the rewards and the financial, medical and personal consequences of RTW.

*"“… suppose I suffer a recurrence now, then I will receive half of three days from National Insurance; then you start weighing it up…”"*[20]

They mused on whether it was worth the effort to return to work, taking into account their future and uncertain health. The rewards might count for little, compared to the efforts, as the interviewees expressed.

*"“… when I go to work, I won’t have any more money, and I will never be able to handle full-time anymore (…) so either I go to work or I don’t, I earn nothing…no, to me that doesn’t matter…”"*[12]

*"“… and that’s the difference between working and earning, my husband also says, ‘Are you going to bring all that on you again, for that small difference?’ But it isn’t just the financial part, there’s something missing in my life…”"*[1]

These quotations show that the financial implications of (part-time) RTW or of recurrence were considered. Although the women looked forward to being an employee again, they also reconsidered the financial and personal need to return to work.

*"“… I was 47 when it had happened. When you are 50, you profit a lot more from a career break, but you don’t get that when you’re not yet 50, so that worried me a bit, yes, the financial part does matter…”"*[20]

This woman, for instance, wished to spend more time as a mother, at least temporarily in the near future.

*"“… I also don’t want to work full-time anymore, not right now, I also want do things here, I also want time together with X (daughter), in this I have indeed been shaken up…”"*[13]

#### Concerned about recovery

A third group of considerations was about capability and the women’s doubts about performing as an employee again. One of the women’s questions was whether it was wise, from a medical point of view, to return to work: working might be too exhausting and strenuous. The feeling of being capable of working again might change from one day to the next.

*"“… some days I’ll say: yes, I can handle it and I want to go and do something, but the next day I really feel awful again…and then you think, what if I went to work now and I felt like that, then what would you do…”"*[12]

The women wanted to be sure of being ready for RTW; in that way, they would feel as if there were fewer obstacles to RTW. However, some mentioned they had to struggle and try to accept that the desire to return to work was no longer realistic. This was reported by an employee working in a children’s home:

*"“… I really wanted to, but it just wasn’t possible anymore. I think that’s when I realized: it hurt. Every time I went there I thought: ‘I can’t do my job anymore, I have to believe what the doctors say’. I noticed when I was keeping house that I couldn’t do normal things and I thought: ‘If I can’t do it here, how can I do it there’. That wouldn’t work and I think that was why I had already distanced myself (…) actually I’ve always known it in my mind, but not in my heart…”"*[6]

While indicating their doubts about performing as an employee again, the women did in fact try to define their capability level. Many of them still had mental or physical complaints, which intensified the feeling that they would not be able to manage their appointed tasks. They did not expect to be able to stay the course and some were even convinced of that, although this was difficult to explain to others. Moreover, the women feared recurrence.

*"“… the outsider thinks, she’s tired, but look at her, people don’t know…and it’s not that you are tired and by resting, it goes away, it’s a different kind of fatigue to that perceived by an outsider. It keeps going, it keeps lingering, it pushes you down…”"*[4]

#### Doubts about acceptance in the workplace

Fourthly, the women wondered whether the working environment would really understand their situation after returning. In the case of enduring medical symptoms due to breast cancer they felt it unlikely that 100 % performance would be possible.

*"“… I couldn’t just go to work a little bit, I could do the easy stuff, that wouldn’t be of much use to her, I couldn’t saddle her with all the difficult work (…) you get confronted with the dilemma: if you want to work, you must immediately be able to handle everything, and then you’ll get your full job responsibility back…”"*[17]

The interviewed women expressed their feelings: they weighed up their work capacity and their motivation to return to work against their knowledge of their workplace, or their employer.

*"“… at this time I don’t see myself going back; if it was only for sales, or if they’d say: you’ll get a desk job, you can calculate the prices, or enter data in the computer, something like that, then I would be back there tomorrow…”"*[4]

The women commented on how they felt. Some simply feared their employer and painted him or her in a poor light. They argued that they had to be especially strong and in good health to feel able to return to work.

*"“… she made it very clear: I must not think that when I return, she will say you only have to do this or that, because then your colleagues will have to take over all the hard work and that wouldn’t be fair to them (…) But if she’s forced to take me back now, then I won’t survive another month, because she’ll be bullying me and she’ll make me work all the holidays, all the weekends, etc, and give me hard work on purpose, etc, so (…) yes, that’s what I believe, I’m almost certain of that…” "*[5]

All these considerations demonstrate how intensely breast cancer patients prepared for RTW and reflected on their current and future situation.

### Emotional process

These inner reflections were accompanied by various strong emotions. The fear of recurrence gave particular cause for anxiety. The women felt as if they had been “beaten with a sledge hammer” [participant 8], when they were diagnosed with breast cancer, and they still remembered that feeling. They wanted to keep a level head, but this heavy blow seemed to be still on their mind Table 3.

**Table 3:** Themes and subthemes regarding the emotional process

**Themes**		**Subthemes**
Anxiety	·	Fear of death
	·	Fearing recurrence
Fear	·	Financial and medical insecurity
	·	Insecurity about the future
Frustration	·	Not being able to perform as usual
	·	Feeling powerless
Anger	·	Assuming one is not welcome at work
	·	Expecting recognition and support

*"“… there’s always something hanging over your head…”"*[17]

It was a fear they had to learn to live with, as this woman described, because it took up a lot of space in their lives (and in their head).

*"“… yes, it’s somehow also a realisation of the fact that you really have to start living a bit for yourself again, and on the other hand of course you are left with an enormous fear, and it still hasn’t been dealt with. I believe that you’re never truly free of that, because it remains in the back of your head a bit…”"*[14]

Not knowing whether one would recover completely, or expecting to be unable to do one’s work brought with it feelings of fear as well as an enormous (financial) insecurity about the future. The women might feel frustrated and scared.

*"“… you know that you can’t really handle the work anymore, you don’t know how your life is going to look further down the line and that’s frightening…”"*[12]

Some told us that they felt really powerless to manage their insecurity and fear of not being able to work as usual. One woman did not want to feel guilty about not being able to perform as usual and tried to find a new, but ‘faceless’ job where she could work ‘anonymously’.

*"“… I’ll just go somewhere they don’t know me, where I’m just a number and if anything goes wrong then I won’t have to take it to heart when I fall short of the mark…with regard to X (previous employer) I felt guilty that I couldn’t be there anymore…”"*[13]

A few women expressed the fear that their employer might not eagerly welcome them back and this might lead to distressing anger.

*"“… (CT: if the advisory physician says: you are capable)…then I will just return to work (…) then I’m afraid that she (employer) is going to give me such a hard time that I’m going to say, I won’t come anymore, but I’d never do that. I’ve worked so many years for my retirement; I’m not going to allow that to be taken away from me, that’s what I’m afraid of…”"*[5]

If they expected recognition and support from the work environment, women had the courage to trust the final RTW outcome, which gave them a feeling of rest and peace.

*"“… they know what I had to deal with, so they take that into account and so it’s not that you have to work really hard for 8 hours straight; when there are customers, you have to attend to them; if nobody’s there, you can sit down, so (…) if I hadn’t done this reconstruction, I would have returned to work a long time ago…”"*[16]

These intense emotions, apparent from the women’s (non-verbal) reactions, were very convincing to the author who conducted the interviews. Many women spoke softly and fell silent for a while or were moved to tears. We even had to arrange aftercare for one of the women.

### Interaction with the social environment

The considerations were clearly related to the various messages from the social environment. As the participants mentioned, their inner thoughts and feelings concerning the (possible) work resumption were shared with the work environment, the insurance, medical, and private environment, and these interactions influenced their mental preparation for RTW Table 4.

**Table 4:** Themes and subthemes regarding the interaction with the social environment

**Themes**		**Subthemes**
Work environment	·	Doubts regarding the employee’s condition and ability
	·	Not knowing how to support the employee
	·	No understanding of or encouraging RTW
Insurance environment	·	Obligation to return to work sooner than expected (by the employee)
	·	Feeling pressured and misunderstood
Medical environment	·	Advising against RTW for medical reasons
	·	Protecting the employee from returning to work too soon
Private environment	·	Discussing the considerations regarding RTW
	·	Advising against RTW in case of problematic reintegration

The interactions with the work environment were closely linked to the women’s concerns about their own capability and about their doubts regarding acceptance within the workplace. The interviewees noticed that the employers’ doubts could appear on different levels for various reasons. The employer could, for example, doubt the employees’ abilities and gave that as a reason for not wanting to place an extra work load on the employees’ colleagues.

*"“… for the brief space of time that I go, even though I only do it half-time, for 15 or 20 hours, he insists that in the period that I’m there, he can count on me for 100 percent (…) there’s no adaptation, it’s not such a large company, everyone does everything there and is supposed to do everything there and he said that ‘the colleagues can’t keep taking on your work’…”"*[4]

In some cases the employer might pretend to be protective by advising the employee to continue on sick leave, because she did not know how to ‘design’ and prepare the return, taking into consideration the employee’s condition.

*"“… I rang up to tell them: I’m coming back, I’m doing well, and then she said: ‘yes, but are you able to handle your job again, because there’s no other job available’ (…) Now I’m still on sick leave, because the employer said that it is better that I remain ill for a while, because she doesn’t have a new job for me (…) that she wants to protect me a bit from being fired again, right after I return…” "*[18]

On the one hand, the employer could be quite understanding or encouraging about the intended RTW; on the other he could reveal his doubts about RTW or not (immediately) endorse the plan to return. Women felt that employers were understanding, if they were allowed to test their abilities.

*"“… I asked if I could come back, and ‘yes of course you can come back’ and I say: ‘can I come back and work half my hours and then see?’, and they said ‘of course’, that was all fine ‘you just try and see what you can handle’…”"*[15]

Whether women felt able and sufficiently recovered to return to work was strongly influenced by the employer’s attitude. This woman responded firmly to her employer’s negative advice:

*"“… but on the other hand I also feel like, you shouldn’t go back somewhere where it has clearly been said that they don’t really want you there…”"*[18]

Interactions with the insurance environment could sometimes lead to mixed feelings, as many women showed. Although most women wanted to return to work as soon as possible, some were advised by the insurance physician to go back to part-time work sooner than they wanted. In that case the women mentioned that they felt pressured and misunderstood if they considered themselves not to be fully recovered. However, other people seemed to think they were back to normal.

*"“… they’re really harsh, I told them a few times that I just couldn’t handle it mentally, but they just sent me to work, whether you can handle it or not, I think you should say then: I’ll jump off the bridge here, maybe then they’d realise, but otherwise, no, it’s that I find so hard. How can you say that you feel miserable, that you don’t feel well? You’re looking fine (raising her voice) and then they think she is feeling fine, that’s fine, but they should start thinking about how profound this can sometimes be (tears)…”"*[12]

According to a few women, treating oncologists (and other practitioners) would advise against RTW for medical reasons. Practitioners seemed to assist the employee and protect her from returning to work too soon.

*"“… I went to the doctor with that plan and said what I wanted and the first oncologist said: madam, I see that you’re really willing to try…and that’s okay with me. I’m not going to stop you, if you really want to try it, but not yet (…) I see that you really want to, he said, I can’t stop you, but that’s what I’m telling you, and you probably won’t like hearing it, but I am going to stick with what I believe…”"*[6]

As we have seen in the case of the employer and the insurance physician, the women could receive conflicting messages. This also applied to the medical physician and insurance companies. These conflicting messages raised women’s concerns and dependence, as witnessed below.

*"“… I was tired, I was lying down every day, I was really sleeping; naturally, your body adjusts to that, but the doctors say: ‘You must do that’, supervision says: ‘Then you should go for a walk, gradually you’ll build it up again’ but the RVA (governmental department for employment provision) says: ‘yes, you have to go to work, there’s nothing wrong with you anymore’ and the people at National Insurance say: ‘what exactly is the problem’ because they don’t see anything wrong with you…”"*[13]

The women also discussed their considerations regarding RTW at home. After a long period of disability, some women became used to a slower pace of life and thoughts of resuming work were accompanied by continuous discussion with their family.

*"“… I wanted to keep that peace (…) I’ve really been whining to my husband for three months, should I go back, should I not go back, could we manage without my wages and so on, and he says: ‘I don’t think you have to return to work, as far as I’m concerned you can stay at home’ and…maybe I’m going to regret it afterwards…I’ve really been going on about it…”"*[11]

The influence from the personal environment seemed especially important if the return did not go smoothly or if the woman awaited and weighed up her own RTW. The partner’s advice could be of overriding importance, especially when the women felt they had to make a stand against the employer.

*"“… when I think about it, my head starts swimming, it’s not that I’m lazy or anything. My husband says: 'you shouldn’t take it to heart, don’t keep thinking about it, if you have to go back and she (CT: the employer) doesn’t want you, then you can just stay home for those few years, we’ll make do with less' (…) it’s not that I, how should I say this, I don’t miss work, (…) I really don’t miss it (…) but I sometimes feel like: I also have to contribute my bit…”"*[5]

To recapitulate, the various considerations accompanied by strong emotions were not only an issue for the women themselves but were also a reflection of the social environment. The employer’s attitude seemed of particular importance.

### Preparation permeated by uncertainty and vulnerability

During the mental preparation for work, strong emotions could emerge. Most women had a particularly genuine ambivalence about the impending and future (work) role. First of all, they felt unsure and vulnerable because they could never be sure if they had completely survived. As many women said: they always feared recurrence and were aware of their mortality. Secondly, they mentioned that they did not know how to do their job in a positive and valuable way. In the light of their ability and the employer’s understanding nearly all women doubted whether they would be accepted again in the working environment.

*"“… They told me, you really can’t bathe a 100 kilogram person anymore, the job you have done you can’t do that anymore. I cannot manage all that, I still need help so how can I go help other people when I can’t even help myself, so I’ll just wait. Gosh, what a lot of help is necessary to be able to return to work again, you can’t go to work when you yourself.... Emotionally, things are better now, but that arm like I said, that doesn’t work, so in the first place it’s something physical, and then psychologically, you still have fits of crying, you’re still sensitive…”"*[2]

Their vulnerability was about being unsure whether they would survive in the future and about the vagueness of their ability. Their uncertainty referred to the lack of clarity about work resumption and what the employer would allow. This vulnerability and uncertainty was felt during the whole process. Even though they felt self-confident and able to return, most women had doubts and deep thoughts. Some felt like an outsider in society, powerless to do anything about it.

*"“… but when she spoke about it (RTW) I always got the feeling that she silenced me, saying I wasn’t able to handle it, and you keep feeling that powerlessness (…) I want to go back, but your body doesn’t feel like you can handle things properly again, you don’t feel capable of participating in society, because in this fast society expectations run much too high…”"*[17]

Degrees of vulnerability and uncertainty largely depended on the dynamic interactions with the social environment (work, insurance, medical, and private) especially in the case of conflicting messages.

*"“… and every time I go there they say, you won’t manage and it’s getting much busier, you won’t manage anymore, so she doesn’t encourage me to start working again, she’s always opposing, she really is a difficult person…”"*[5]

Despite their vulnerability or uncertainty, most women did not feel that they were failures or objects of pity.

*"“… I can still do things, and I really want to contribute the things that I can still do, but it can’t be done at the pace that society expects and that’s where there should be some sort of side-track (…) but that doesn’t exist anymore, because nowadays they won’t say to you, just come and do the easy work for the same amount of money, because then your colleagues will object because solidarity is not what it used to be (…) now it’s everyone for himself and God for all of us…” "*[17]

Despite their ambivalence about the future, it was clear that the women preferred to return to work, but only on condition that it would be adapted to their new situation.

## Discussion

To answer the research question about how Flemish women who have undergone breast cancer surgery and reported sick, mentally prepare for return to work, we interviewed 22 employees, on average two and a half years after breast cancer surgery. We found that once their treatment finishes, women start to mentally prepare their return and intensively reflect on their current and future situation. Four different matters are considered prior to RTW: 1) leaving the sick role and wanting to keep the job; 2) doubting whether working is worth the effort; 3) doubting their capability; and 4) doubting the acceptance from the workplace after returning. These inner reflections are accompanied by strong emotions. They are developed and affected by interactions with important actors from their social environment, especially the employer. The whole process is coloured by uncertainty and vulnerability.

### Study adds to what is already known

There is still very little known about women’s concerns regarding work after breast cancer treatment, but our findings seem to be in line with earlier findings and clearly add to our understanding of the process of preparing RTW. From quantitative studies we know that the women’s decision about returning to work is negatively affected by several aspects of mental preparation: uncertainty about ability to work; uncertainty about possible job loss; and health and work characteristics on the women’s decision about returning to work [22-24]. Our study shows how this mental process is shaped and how the women intensively reflect on their situation prior to RTW.

Quantitative studies do not agree on the effects of physical problems on RTW. Fantoni et al [17] showed that not the physical, but the psychological concerns impact the time until return to work [17]. Oberst et al [25] however found physical problems to be more problematic for RTW than cognitive impairments for employees with either breast or prostate cancer [25]. According to Munir et al [26] the women’s appraisal of their ability to manage work tasks is influenced by (1) actual cognitive ability after chemotherapy, (2) awareness of cognitive failures, and (3) impact on their confidence in carrying out work tasks [26]. Another factor that might elicit concern was demonstrated by Cooper et al [27]. They found that some women with breast cancer might feel pressured to return to work too soon because of financial concerns [27]. Our study indeed demonstrated the interrelatedness of the wish to return to work, the doubts about whether work is worth the effort and about physical and mental capacity and acceptance from the workplace. We have shown that the women have these inner reflections at the same time and that these reflections yield contradictory answers regarding the question of whether the woman wants to return to work. Women thus have ‘mixed feelings’ regarding RTW. However, quantitative research seems unable to grasp this ambiguity.

In a recent qualitative research by Tamminga et al [28] a large variety of concerns regarding health and RTW of women with breast cancer is also found. Since they conducted a content analysis, focusing on describing the process rather than developing a new theory, they did not prioritise concerns nor analyse their interrelatedness [28]. According to other authors [29,30] women wish to return to the labour market after illness, which is regarded as a sign of returning to life in spite of concerns about their physical problems. In the current study we also noticed the women’s wish to leave the sick role and keep their job.

Previous studies do not deal with the ambiguity regarding RTW that woman with breast cancer experience. Few studies focus on uncertainty and concerns but none of the studies on RTW of breast cancer patients reveal the experienced vulnerability. This is a general picture of RTW research, including that dealing with other illnesses. Recently Stewart et al [31] found five categories of expectations of RTW for injured workers with back pain. All these expectations relate to uncertainty: (1) perceived lack of control over the RTW process; (2) perceived lack of recognition by others of the impact of the injury; (3) perceived inability to perform the former job; (4) the fear of re-injury, and (5) the perceived need for workplace adaptations. This study was able to demonstrate the negative effects of employees’ perceived uncertainty regarding ‘active coping’ with the illness and RTW having a back injury [31]. In their qualitative research of breast cancer patients, Repass and Matusitz [32] found uncertainty to be a central concept of the recovery phase. The women feared recurrence and being socially stigmatized. According to the authors, empowerment is needed by rebuilding healthy lifestyles through physical activity, to regain confidence through the realisation of the return to normality [32]. Women who have breast cancer seem to make a kind of transition from patient to survivor [33,34]. Despite fearing recurrence some employees felt empowered by surviving the experience of breast cancer and made several life changes such as cutting down their work hours [34].

In our study, vulnerability appeared to be an overarching, central concept regarding the mental preparation for RTW and has the potential to be used in RTW research in general. As the current research shows, the concept of vulnerability can be understood in two different ways: being vulnerable (individualised) or being made vulnerable (socialised). Further investigation is needed to elucidate this proposition.

Research on RTW focuses more on self-efficacy, a linked but narrower concept. Self-efficacy refers to people's beliefs about their capabilities [35]. Relationships between high self-efficacy and (earlier) RTW are found in cardiac patients [36] and workers with back pain [37]. Loh et al [38] found positive experiences of self-efficacy in the post-treatment phase of breast cancer survivors. Self-efficacy is an important concept in the motivational models used for studying RTW. The women’s mental preparation for RTW can be regarded as a ‘contemplation’ phase which is considered in the Readiness for RTW model [8]. In the ‘contemplation’ phase a person is beginning to consider RTW somewhere in the future, thinking of the pros and cons and feeling ambivalent, but unable to initiate change. Several dimensions are involved in making progress during the various phases (precontemplation, contemplation, preparation for action, action and maintenance). Decisional balance, the first dimension, reflects the (cognitive) process of weighing up the pros and cons. The second dimension ‘self-efficacy’ refers to one’s confidence in engaging in RTW and activities maintaining RTW’. Thirdly, change processes are assumed to be ‘experiential’ (perceived need to change thoughts, feelings and attitudes including communication with others) and ‘behavioural’ (actual change, e.g. contacting the employer) ([8], p.237).

Our study demonstrated a more complex picture. Women consider pros and cons, but the weighing-up process is ambiguous and confusing and not as rational as the Readiness for RTW model [8] assumes. The women experience increased self-efficacy as they feel recovered and motivated, but at the same time they experience vulnerability and also feel dependent upon their social (particularly work) environment. Regarding the third dimension we did indeed find that some women contacted their employer as regards to RTW, but not as a result of a behavioural change. Another contraction to this model is that our study demonstrated that mental preparation is not a linear process of improvement. The women did not report that they felt more ready for RTW in due course. Our findings show that the women are indeed motivated to take up their professional activities, but feel vulnerable after recovery and in need of some understanding, and this interaction with the environment is not found in the different phase models.

Several theoretical models for RTW [7-10] focus on conditional (behavioural) steps in the phases before RTW. Not following these steps is called recurrence or relapse. This conditional character of the Readiness for Change model [10] has been criticised earlier [28]. Another important point on which our study’s results add to these models regards emotions. We found strong emotions embedded in patients’ preparation for RTW. This might relate to the specific case of breast cancer; these women probably need another approach than just taking action or improving motivation. The experience of having cancer is traumatic and stress-related [33,39]. However, many experiences that were surrounded by emotions addressed issues that will also be experienced by other employees that prepare for RTW after major illness: leaving the sick-role and wanting to keep their job; considering pros and cons of returning to work; (financial) insecurity about the future; worrying about reactions from the workplace, as well as searching for strength and expecting support. Bowles [40] tried to add negative emotions and developed the Adaptive Change model, which is regarded as an improvement to the Readiness for Change model [10]. However, Bowles [40] still conceptualises the preparation for RTW as a linear process and negative emotions are regarded as a barrier to improvement. According to Bowles ([40] p.442) individuals can move more easily from planning to action “if they manage negative emotions, have inner drive, and have social support from others”. Although we did not explicitly analyse the effects of the emotions on RTW rate, our study suggests that emotions are a self-evident part of the process and not a particular barrier.

Our study might point to an issue that is much broader and to another conceptual level that the behavioural models cited above. The issue is that uncertainty and vulnerability might be characteristic for the experience of many employees who prepare for RTW after recovery from a major illness and that thoughts are often confusing rather than a reflection of a linear development towards RTW. This seems to be neglected in mainstream research on RTW. The current discourse (in particular in activating RTW programmes) emphasises self-management, empowerment (connected to expectations and achieving goals), self-responsibility et cetera e.g. [9,32,34,40,41], but as a result might neglect other important experiences that point to issues of confusion, uncertainty and vulnerability. Using life narratives Van Hal et al [41] demonstrated that work disability “changed [the life] to such an extent that life cannot be lived as it had been before” ([41], p. 83). In-depth qualitative research among people on long-term disability benefit in the Netherlands revealed that a “pending process of identity work” ([42], p. 89) takes place, which means that (injured) persons have to think about how to relate to their past, present and future, and search for a new basis in life. During this process they wish to be heard and supported.

Furthermore, the models discussed above seem to have a too individualistic focus, while the social environment in its broad sense also constitutes an important influence on the RTW process. Several studies, both qualitative and quantitative, demonstrate a lack of understanding from the work environment [28,43,44]. Our study also demonstrates the importance of the social environment not only in terms of expected support but also in terms of the institutional environment. Most women in our study do not know prior to RTW whether they are allowed to adapt their employment contract. This is because, in Belgium, there is no legislation forcing employers to guide RTW [39]. This might explain why the interviewed women on the one hand made strong statements regarding their wish to return to work and at the same time expressed feelings of dependence, vulnerability and uncertainty. The Belgian legislation not only seems to lead to a lack of support [39] but also seems to reflect the implicit norm that employees are expected to handle their absence from work alone and take the RTW initiative themselves. This effect of the institutional context is different from what is distinguished as the interpersonal context in the Readiness for RTW model [8].

### Study limitations

We only interviewed 22 women in the Flemish (Belgian) context, which is a limitation in relation to generalising the results. Furthermore, we do not know why almost two thirds of the invited women decided not to participate in our study, which is a selection bias. However, earlier we described the various experiences we found of being work disabled after breast cancer treatment [15]. The current analyses are conducted in the same sample. In the face of these largely different experiences of work disability, we now discover that women feel similar when preparing for their RTW. They all have inner reflections concerning RTW and feel vulnerable and uncertain about RTW, which makes them dependent on the medical, insurance, private and work environment, to some extent. The variety in the earlier analyses and the similarity of experiences in this study contribute to the transferability of our findings.

## Conclusions

Our findings show a detailed picture of four types of considerations made by breast cancer survivors before they actually resume work. In future research on breast cancer patients on sick leave, these considerations plus the accompanying emotions and the role of the social environment should not be ignored. It is also necessary to study the mental consideration, emotions and attitudes of the social environment in relation to actual RTW.

Knowledge of the mental considerations might help to better support women with breast cancer. We illustrated that (preparing for) RTW for breast cancer survivors is a complex process, characterised by vulnerability, and not a simple (addition) sum of factors, dimensions and forward or backward phases. We argue that the findings also have implications for theoretical models for RTW that seem to have a focus that is too rationalistic, linear and individualistic and might therefore miss an important part of reality.

The practical implications for professionals are that they should take into account the feeling of vulnerability of breast cancer survivors. They are advised to understand the women’s precarious situation for which they cannot be blamed and to offer them fulfilling work adapted to their capabilities, so they can increase their self-efficacy and gain positive experiences and confidence during RTW. The environment has to be aware of the possibility of decreasing or increasing women’s vulnerability and uncertainty while preparing for RTW. We found that some women feel pressured by their environment to return to work, which indeed made them more vulnerable. Vulnerability might also be a risk factor for depression after RTW [45].

## Competing interests

The authors indicated no potential conflicts of interest.

## Authors’ contributions

All authors participated in design, analysis and interpretation of the data. All authors contributed to the content of this manuscript, commented on drafts or revised it critically. All authors read and approved the final manuscript.

## Pre-publication history

The pre-publication history for this paper can be accessed here:

http://www.biomedcentral.com/1471-2458/12/538/prepub

## References

[B1] Spelten ER Sprangers MAG Verbeek JHAM Factors reported to influence the return to work of cancer survivors: a literature review Psycho-Oncol 2002 11 124 131 10.1002/pon.585 11921328

[B2] Renard F Van Eyken L Arbyn M High burden of breast cancer in Belgium: Recent trends in incidence (1999–2006) and historical trends in mortality (1954–2006) Archives of Public Health 2011 69 2 10.1186/0778-7367-69-2 22958447 PMC3436615

[B3] Johnsson A Fornander T Rutqvisk L-E Predictors of return to work ten months after primary breast cancer surgery Acta Oncol 2009 48 93 98 18937082 10.1080/02841860802477899

[B4] Roelen CA Koopmans PC de Graaf JH Balak F Groothoff JW Sickness absence and return to work rates in women with breast cancer Int Arch Occup Environ Health 2009 82 543 546 18795317 10.1007/s00420-008-0359-4

[B5] Balak F Roelen C Koopman P Ten Berge E Groothof J Return to work after early-stage breast cancer: A cohort study into the effects of treatment and cancer-related symptoms J Occup Rehab 2008 18 267 272 10.1007/s10926-008-9146-z 18670868

[B6] Eaker S Wigertz A Lambert PC Bergkvist L Ahlgren J Lambe M Breast cancer, sickness absence, income and marital status. A study on life situation 1 year prior diagnosis compared to 3 and 5 years after diagnosis PLoS One 2011 6 3 e18040 www.plosone.org 21479209 10.1371/journal.pone.0018040 PMC3068139

[B7] Krause N Ragland DR Occupational disability due to low back pain: A new interdisciplinary classification based on a phase model of disability Spine 1994 19 1011 1020 8029734 10.1097/00007632-199405000-00002

[B8] Franche RL Krause N Readiness for Return to Work following Injury or Illness: Conceptualizing the Interpersonal Impact of Health Care, Workplace, and Insurance Factors J Occup Rehab 2002 12 233 256 10.1023/a:1020270407044 12389476

[B9] De Rijk A Janssen N Van Lierop K Alexanderson K Nijhuis F A behavioural approach to RTW after sickness absence: The development of instruments for the assessment of motivational determinants, motivation and key actors’ attitudes Work 2009 33 273 285 19759426 10.3233/WOR-2009-0875

[B10] Prochaska JO DiClemente CC Norcros JC In search of how people change Am Psychol 1992 47 1102 1114 1329589 10.1037//0003-066x.47.9.1102

[B11] Arora NK Rutten LJ Gustafson DH Moser R Hawkins RP Perceived helpfulness and impact of social support provided by family, friends, and health care providers to women newly diagnosed with breast cancer Psycho-Oncol 2007 16 474 486 10.1002/pon.1084 16986172

[B12] Al-Azri M Al-Awizi H Al-Moundhri M Coping with a diagnosis of breast cancer: literature review and implications for developing countries Breast J 2009 15 615 622 19686231 10.1111/j.1524-4741.2009.00812.x

[B13] Doumit M El Saghir N Huijer H Kelley J Nassar N Living with breast cancer, a Lebanese experience Eur J Oncol Nurs 2010 14 42 48 19815459 10.1016/j.ejon.2009.08.003

[B14] Drageset S Lindstrøm TC Underlid K Coping with breast cancer: between diagnosis and surgery J Adv Nurs 2010 66 149 158 20423441 10.1111/j.1365-2648.2009.05210.x

[B15] Tiedtke CM Dierckx de Casterlé B de Rijk A Christiaens MR Donceel P Breast cancer treatment and work disability: Patient perspectives Breast 2011 10.1016/j.breast.2011.06.002 21723729

[B16] Salander P Liliehorn S Hamberg K Kero A The impact of breast cancer on living everyday life 4.5-5 years post diagnosis – a qualitative prospective study of 39 women Acta Oncol 2011 50 399 407 21395468 10.3109/0284186X.2010.547216

[B17] Fantoni S Peugniez C Duhamel A Skrzypczak J Frimat P Leroyer A Factors related to Return to work by women with breast cancer in Northern France J Occup Rehab 2010 20 49 58 10.1007/s10926-009-9215-y 19902340

[B18] Tighe M Molassiotis A Morris J Richardson J Coping, meaning and symptom experience: a narrative approach to the overwhelming impacts of breast cancer in the first year following diagnosis Eur J Onc Nurs 2011 10.1016/j.ejon.2011.03.004 21511530

[B19] Anema JR Schellart AJ Cassidy JD Loisel P Veerman TJ Van der Beek AJ Can cross country differences in return-to-work after chronic occupational back pain be explained? An exploratory analysis on disability policies in a six country cohort study J Occup Rehabil 2009 19 419 26 19760488 10.1007/s10926-009-9202-3 PMC2775112

[B20] Corbin J Strauss A Basics of Qualitative Research 3e: Techniques and Procedures for Developing Grounded Theory 2008 Sage Publications, Los Angeles, London, New Delhi, Singapore

[B21] Dierckx De Casterlé B Gastmans C Bryon E Denier Y QUAGOL: A guide for qualitative data analysis Int J Nurs Stud 2011 10.1016/j.ijnurstu.2011.09.012 21996649

[B22] Tiedtke C De Rijk A Dierckx de Casterlé B Christiaens MR Donceel P Experiences and concerns about ‘returning to work’ for women breast cancer survivors: a literature review Psycho-Oncol 2010 19 677 638 10.1002/pon.1633 19823971

[B23] Feuerstein M Todd B Moskowitz M Bruns G Stoler M Nassif T Yu X Work in cancer survivors: a model for practice and research J Cancer Surviv 2010 4 415 437 20945110 10.1007/s11764-010-0154-6

[B24] Campbell KL Pusic AL Zucker DS Mc Neely ML Binkley JM Cheville AL Harwood KJ A prospective model of care for breast cancer rehabilitation: Function Cancer 2012 118 2300 2311 10.1002/cncr.27464 22488704

[B25] Oberst K Bradley CJ Gardiner JC Schenk M Given CW Work task disability in employed breast and prostate cancer patients J Cancer Surviv 2010 4 322 30 20549572 10.1007/s11764-010-0128-8

[B26] Munir F Burrows J Yarker J Kalawsky K Bains M Women’s perceptions of chemotherapy-induced cognitive side affects on work disability: a focus group study J Clin Nurs 2010 19 1326 1370 10.1111/j.1365-2702.2009.03006 20500346

[B27] Cooper AF Hankins M Rixon L Eaton E Grunfeld EA Distinct work-related, clinical and psychological factors predict return to work following treatment in four different cancer types Psycho-Oncol 2012 10.1002/pon.3049 22434715

[B28] Tamminga SJ De Boer AG Verbeek JH Frings-Dresen HW Breast cancer survivors’ views of factors that influence the return-to-work process – a qualitative study Scand J Work Environ Health 2011 10.5271/sjweh.3199 21986836

[B29] Kennedy F Haslam C Munir F Pryce J Returning to work following cancer: a qualitative exploratory study into the experience of returning to work following cancer Eur J Cancer Care 2007 16 17 25 10.1111/j.1365-2354.2007.00729.x 17227349

[B30] Johnsson A Fornander T Rutqvist L Olsson M Factors influencing return to work: a narrative study of women treated for breast cancer Eur J Cancer Care 2010 19 317 323 10.1111/j.1365-2354.2008.01043.x 19708931

[B31] Stewart A Polak E Young R Schultz I Injured workers’ construction of expectations of return to work with sub-acute back pain: the role of perceived uncertainty J Occup Rehabil 2012 22 1 14 21656253 10.1007/s10926-011-9312-6

[B32] Repass M Matusitz J Problematic Integration Theory: Implications of supportive communication for breast cancer patients Health Care Women Int 2010 31 402 420 20390662 10.1080/07399330903359326

[B33] Garofalo JP Choppala S Hamann HA Gjerde J Uncertainty during the transition from cancer patient to survivor Cancer Nurs 2009 32 E8 E14 19444082 10.1097/NCC.0b013e31819f1aab PMC3682664

[B34] Allen JD Savadatti S Gurmankin Levy A The transition from breast cancer ‘patient’ to ‘survivor’ Psycho-Oncol 2009 18 71 78 10.1002/pon.1380 18613299

[B35] Bandura A Ramachaudran VS Self-efficacy Encyclopedia of human behavior Volume 4 1994) Academic Press, New York 71 81 (Reprinted in H. Friedman [Ed.], Encyclopedia of mental health. San Diego: Academic Press, 1998). [http://www.des.emory.edu/mfp/BanEncy.html]

[B36] Fitzgerald S Becker D Celentano D Swank R Brinker J Return to work after percutaneous transluminal coronary angioplasty Am J Cardiol 1989 68 1108 12 2816763 10.1016/0002-9149(89)90861-8

[B37] Richard S Dionne C Nouwen A Self-efficacy and health locus of control: relationship to occupational disability among workers with back pain J Occup Rehab 2011 2 421 30 10.1007/s10926-011-9285-5 21279425

[B38] Loh S Ong L Ng L-L Chew S-L Lee S-Y Boniface G Qualitative experiences of breast cancer survivors on a self-management intervention: 2 year post-intervention Asian Pacific J Canc Prev 2011 12 1489 1495 22126487

[B39] Tiedtke C Donceel P Knops L Désiron H Dierckx de Casterlé B De Rijk A Supporting return-to-work in the face of legislation: stakeholders’ experiences with return-to-work after breast cancer in Belgium J Occup Rehabil 2011 10.1007/s10926-011-9342-0 22105670

[B40] Bowles TV The adaptive change model: an advance on the transtheoretical model of change J Psychol 2006 140 439 457 17066751 10.3200/JRLP.140.5.439-457

[B41] Van Hal L Meershoek A Nijhuis F Horstman K The ‘empowered client’ in vocational rehabilitation: the excluding impact of inclusive strategies Health Care Anal 2011 10.1007/s10728-011-0182-z PMC3400028 21755291

[B42] Van Hal L Meershoek A De Rijk A Nijhuis F Going beyond vocational rehabilitation as training of skills: return-to-work as an identity issue Disabil & Soc 2011 1 81 93

[B43] Grunfeld EA Low E Cooper AF Cancer survivors' and employers' perceptions of working following cancer treatment Occup Med 2010 60 611 7 10.1093/occmed/kqq143 20855546

[B44] Banning M Employment and breast cancer: a meta-ethnography Eur J Cancer Care 2011 20 708 719 10.1111/j.1365-2354.2011.01291.x 21933291

[B45] Østergaard D Dalton SO Bidstrup PE Poulsen AH Frederiksen K Eplov LF Johansen C Mortensen EL Mental vulnerability as a risk factor for depression: A prospective cohort study in Denmark Int J Soc Psychiatry 2011 10.1177/0020764010396409 21441278

